# DDoS attack detection in smart grid network using reconstructive machine learning models

**DOI:** 10.7717/peerj-cs.1784

**Published:** 2024-01-09

**Authors:** Sardar Shan Ali Naqvi, Yuancheng Li, Muhammad Uzair

**Affiliations:** 1 School of Control and Computer Engineering, North China Electric Power University, Beijing, China; 2 Department of Computer Engineering, COMSATS Institute Of Information Technology, Wah cantt, Pakistan

**Keywords:** Cyber security, DDoS attack detection, Smart grid, Auto-encoder, Reconstructive machine learning, Smart Grid protection, Intrusion detection, Threat mitigation, Extreme learning machine (ELM) autoencoder, Deep auto-encoder

## Abstract

Network attacks pose a significant challenge for smart grid networks, mainly due to the existence of several multi-directional communication devices coupling consumers to the grid. One of the network attacks that can affect the smart grid is the distributed denial of service (DDoS), where numerous compromised communication devices/nodes of the grid flood the smart grid network with false data and requests, leading to disruptions in smart meters, data servers, and the state estimator, ultimately effecting the services for end-users. Machine learning-based strategies show distinctive benefits in resolving the challenge of securing the network from DDoS attacks. Regardless, a notable hindrance in deploying machine learning-based techniques is the requirement of model retraining whenever new attack classes arise. Practically, disrupting the normal operations of smart grid is really discouraged. To handle this challenge effectively and detect DDoS attacks without major disruptions, we propose the deployment of reconstructive deep learning techniques. A primary benefit of our proposed technique is the minimum disruption during the introduction of a new attack class, even after complete deployment. We trained several deep and shallow reconstructive models to get representations for each attack type separately, and we performed attack detection by class-specific reconstruction error-based classification. Our technique experienced rigid evaluation *via* multiple experiments using two well-acknowledged standard databases exclusively for DDoS attacks, including their subsets. Later, we performed a comparative estimation of our outcomes against six methods prevalent within the same domain. Our outcomes reveal that our technique attained higher accuracy, and notably eliminates the requirement of a complete model retraining in the event of the introduction of new attack classes. This method will not only boost the security of smart grid networks but also ensure the stability and reliability of normal operations, protecting the critical infrastructure from ever-evolving network attacks. As smart grid is advancing rapidly, our approach proposes a robust and adaptive way to overcome the continuous challenges posed by network attacks.

## Introduction

A smart grid combines traditional power grid systems and digital networks, enabling two ways of communication between the grid and the end user, power grid stability tracking, remote access to smart power appliances, and updates about consumer behavior. Integration of smart devices, services, protocols, and standards ensured simple and effective power system operations but made smart grid networks vulnerable to cyber-attacks. A cyber-attack has the potential to exert a considerable impact on the smart grid infrastructure and society. Therefore, the binding for robust security measures is essential to secure the reliable operation of the smart grid network. This challenge has appeared as a notable research focus recently. Cyber-attackers target cyber resilience elements like data availability, integrity, and confidentiality to manipulate the data used for the control and operation of smart grid networks to gain financial benefits or to disturb the normal operations of the grid network. Multiple techniques for the prevention of cyber-attacks have undergone rigid analysis and deployment by researchers, to fortify network infrastructure and data against unauthorized intrusion. Mehrdad et al. (2018) systematically categorized cyber-attacks into two types: indirect and direct cyber-attacks. Direct attacks were further categorized into four different groups. Among these groups, intrusion attacks appeared to be the most dominant, with denial-of-service (DoS) attacks being recognized as the most destructive. In DoS attacks, the attacker provides false data to the primary source of services, disrupting the normal legitimate services and operation of the smart grid network. Its advanced form is a distributed denial of service (DDoS) attack, in which the attacker simultaneously targets multiple nodes. Detecting a DDoS attack from a single node becomes challenging because it cannot differentiate between legitimate and illegitimate service requests, often resulting in unsuccessful DDoS attack prevention. smart grid networks can be secured from DDoS attacks by analyzing the patterns of network data. Real-time network data analysis can detect DDoS attacks timely and prevention measures can reduce the damage significantly. Machine learning-based algorithms have been gaining popularity in recent times for their effectiveness in detecting DDoS attacks. Mihoub et al. (2022), Almaraz-Rivera, Perez-Diaz & Cantoral-Ceballos (2022) and Ali & Li (2019) proposed Multiple DDoS attack detection approaches. A prime challenge faced by these techniques is to integrate a new class of attack into a learned model whenever an attacker introduces a new class of attack, which is not practical in run-time scenarios, specifically in smart grid networks, where disruption of operations is expensive and discouraged extensively. We proposed an efficient reconstructive machine learning-based model to resolve this problem. The proposed model allows real-time non-disruptive addition of new attack classes even after full deployment. More specifically, we train multiple shallow and deep reconstructive models to learn a class-wise representation of each attack type extensively and designed a class-specific reconstruction error-based classification approach for DDoS detection in smart grid networks. Our method offers the addition of a new attack class without disrupting the already-learned models. After the introduction of every new attack class, our model needs to learn the only newly added class-specific reconstructive model. We develop our approach by exploiting multilevel deep and shallow reconstructive models for retaining rich features. These include the deep autoencoder (Sindian & Samer, 2020), Stacked de-noising autoencoder (Manan et al., 2023), extreme learning machine autoencoder (Sowparnika et al., 2023), and marginalized stacked denoising autoencoders (Deepa & Sivakumar, 2022). We performed experimental evaluations on two specified benchmark databases for DDoS attacks, including their subsets. Hereafter, we compared the outcomes with six advanced methods. Outcomes demonstrate that our approach surpasses the compared techniques with respect to accuracy while at the same time does not need re-training if new attack classes are introduced. Among diverse reconstructive models, the proposed method proves the most convincing performance for handling the specific problem under consideration.

The following are the main insights of this work:

• To avoid the expensive step of post-deployment system retraining, we propose a reconstructive model-based DDoS detection system. Attack-specific reconstruction-based techniques have not been yet analyzed for DDoS detection problems.
• For the above purpose we design a class-specific reconstruction error-based classification algorithm and study the performance of different shallow and deep reconstructive models in conjunction with our algorithm.
• The proposed method is evaluated using standardized benchmark datasets to evaluate its significance in detecting DDoS attacks within smart grid networks. The outcomes are compared to six DDoS detection methods concerning classification accuracy. Outcomes indicate that our method achieves comparable results while not requiring full model retraining if new class types are introduced after deployment.

The remaining sections of the paper are structured as: In the “Literature Overview” section, a detailed review of the current literature on deep and shallow machine learning-based techniques for detecting DDoS attacks within smart grid networks is presented. The “Proposed Method” section defines a problem statement, delves into the realm of deep learning, and introduces our novel reconstructive-based model designed for classification. Within this section, we provide brief descriptions of essential features, including the deep autoencoder, marginalized stacked denoising autoencoder, and the reconstructive model-based approach to attack classification. Next, we discussed the ensemble model learning and feature encoding scheme used in this work. Next, a complete algorithm employed to detect DDoS attacks in the smart grid network is introduced. The “Experimental Setup” offers an in-depth exploration of the experimental evaluation. Two publicly available datasets were used for testing our model and thereafter showed the results, along with a comparative analysis against previously used methodologies. The “Conclusion and Summary” section contains the conclusive decision and summarizes potential directions for future research in our work.

## Literature Overview

This section explores machine learning-based literature, data sets, real-world applications, and techniques previously used for DDoS detection. Detailed literature analysis is shown in Table 1.

**Table 1:** Existing machine learning techniques and datasets used for DDoS detection.

**Author (Year)**	**Learning method**	**Data set used**
Aktar & Nur (2023)	Deep Contractive Autoencoder (DCAE)	CICIDS2017
	Stochastic threshold method	NSL-KDD
		CICDDoS2019
Ahmed et al. (2023)	Multilayer Perceptron (MLP)	CTU-13 Dataset
		Real Weblogs (Dataset)
Malik, Dutta et al. (2023)	Machine Learning model	IoT-CIDDS
	Cross Validation	
	Hyperparameter Optimization	
Al-Juboori et al. (2023)	eXtreme gradient boosting (XGBoost)	MTM Dataset
	Decision tree	
	Random forest	
	Gradient Boosting	
Assis et al. (2021)	GRU	CICDDoS2019
		CICIDS2018
Banitalebi Dehkordi, Soltanaghaei & Boroujeni (2021)	Classification based algorithm	UNB- ISCX12
	Entropy	CTU-132
	Floodlight controller	
Cil, Yildiz & Buldu (2021)	DNN	CICDDoS2019
Tuan et al. (2020)	Decision tree (DT)	UNBS-NB-15,
	Unsupervised learning (USML)	KDD99
	(K-means, X-means)	
	Support vector machine (SVM)	
	Artificial neural network (ANN)	
Aamir & Zaidi (2019)	kNN, RF models, and SVM	CICIDS2017
Naagas et al. (2018)	DELA	Simulated DDoS
	(Deep Extreme Learning-based autoencoders)	Attacks
Wang et al. (2017)	Honeypot game theory,	AMI data
	Bayesian-Nash equilibrium	
Diovu & Agee (2017)	Cloud-based algorithms	Attack vector,
		AMI traffic
Srikantha & Kundur (2015)	Collaborative reputation-based topology,	MATLAB simulation
	Nash Equilibrium routing topology,	-based data
	Game theory	
Varalakshmi & Thamarai Selvi (2013)	Hierarchical Broker Architecture,	Network traffic data
	Kullback–Leibler Divergence	
Kumar & Selvakumar (2011)	RBP Boost classification algorithm,	KDD Cup,
	Backpropagation,	DARPA 1999,
	Weighted Majority Voting	DARPA 2000

Aktar & Nur (2023) proposed a deep learning model employing a contractive autoencoder, by training model for learning normal traffic patterns from the compacted representation of the input data and applied a stochastic threshold technique for DDoS attack detection. Yaser, Mousa & Hussein (2022) implemented a deep extreme learning machine-based autoencoder for efficiently detecting illegitimate network traffic during a simulated DDoS attack. The hybrid method proposed by Wang et al. (2021) combines a convolutional autoencoder with a long short-term memory (LSTM) network. This fusion actually captures both temporal and spatial features, resulting in improved detection accuracy. Recognizing the potential of various reconstructive machine learning models, Wang et al. (2021) also examined ensemble frameworks to enhance DDoS attack detection further. Drif, Zerrad & Cherifi (2020) implemented the integrated variational autoencoder to further improve the DDoS detection accuracy. Naagas et al. (2018) implemented a multilayered DDoS attack detection system by proposing DELA models. These individual models achieved the maximum sensitivity and adaptability for DDoS attack detection. Mylonas, Abdallah & Chatzi (2021) integrated features from power flow, supervisory control, and SCADA (data acquisition system) with autoencoders, hence further improving the understanding of network dynamics. Liao (2022) used transfer learning for cross-domain implementation, proving that transfer learning is a bridge between distinct domains. Guimaraes et al. (2017) incorporated generative adversarial networks (GANs) and autoencoders to enhance the adversarial robustness and resilience of the model against DDoS attacks. Sadaf & Sultana (2020) used autoencoders for the real-time detection of false data originating from sensors. This focus on real-time capabilities is essential for promptly preventing and detecting DDoS attacks within smart grid networks. Gao et al. (2016) implemented a protocol-independent method based on a reflection approach for DDoS attack detection. Five determined features are used for Machine learning-based DRDoS detection in a protocol-independent manner. The feature set contains multiple attributes within a defined time interval without TCP or UDP headers, like the total packets directed to the target, maximum packets transmitted in a specified time frame, the size of the packet delivered to the target, and the difference between the total packets sent to and received from the target within the specified time frame. Singh, Singh & De (2016) used the naive Bayes-driven classification of machine learning with eight features in consideration of the CAIDA 07 dataset. The naive Bayes method uses statistical formulas to compute probabilities for DDoS attack detection. Azab, Alazab & Aiash (2016) proposed the most powerful feature labeling approach tailored for botnet traffic, directed to as the “Classification of Network Information Flow Analysis” (CONIFA). Their analysis used a botnet toolkit known as Zeus for the objective of traffic generation and succeeding analysis. Yusof et al. (2017) proposed an approach that combines features of DoS with consistency-based subset evaluation (CSE) to determine enhanced features from the subsets of available DDoS datasets, for comparison with traditional feature selection methods based on statistical significance. Singh & De (2017) consider 16 features from the CAIDA’07 dataset to be the most significant, and the proposed ensemble feature selection approach can integrate statistical significance scores, including chi-square, information gain, correlation ranking, reliefF, SVM, gain ratio, and symmetrical uncertainty ranking filters. These scores can be used further to find out a threshold for the exclusion and inclusion of each feature in the finalized feature set, with the threshold being represented as the norm of the unique scores. Khan et al. (2018) presented a novel technique by incorporating entropy with granular computing to form a feature selection method for enhanced DDoS detection. Khan et al. (2018) particularly applied entropy computations to all seven features of the NSL-KDD-09 dataset. Later, each feature is allocated a weight value based on the anomaly count for streamlining the feature selection process. Alejandre, Cortés & Anaya (2017) implemented a wrapper method1 using ISCX and ISOT datasets to choose various feature sets using genetic algorithm and greedy search and then used C4.5 algorithm for evaluation of those features. The features that yield a higher detection rate with the C4.5 are given priority. Al-Hawawreh (2017) discussed the detection of simulated SYN flood attacks studied using various machine-learning techniques. Then the pre-processing and features extraction phase, the addition of an intersection process to select features selection is added to get the optimal feature set on the basis of standard features from gain ratio, information gain, and ReliefF. Li, Liu & Gu (2010) implemented a neural network technique, learning vector quantization (LVQ) on a simulation-based dataset. LVQ produces cluster boundaries under supervised learning according to training data. Neurons exhibiting close relation converge and are eventually grouped jointly within the same class limit *via* multiple iterative techniques. The neurons with the most similar features determine the group class, enforcing the winner-takes-all paradigm. Agrawal et al. (2011) implemented an artificial neural network (ANN) against the divergences in entropy of data, and to find out the strength of a DDoS attack. (Gupta et al. (2011) implemented ANN against the divergences in entropy of data to predict the compromised nodes behind the DDoS attack. Bansal & Mahapatra (2017) detected the illegitimate botnet traffic using three different machine-learning techniques. Initially, a clustering technique is used, clustering with six features called the manual feature extraction with later assigning labels as, botnet, and malicious to every cluster depending on network data observances of specific time frame. Zekri et al. (2017) employed a machine learning approach in the cloud for DDoS detection. A set of five features is examined in this research using the C4.5 decision tree technique. For improved results, the model is complemented with a signature-based technique. For comparison, K-means and naive Bayes, two different approaches are also tested. Yuan, Li & Li (2017) enforced a deep learning-based recurrent neural network (RNN) for the detection of DDoS attacks, and termed it as Deep Defense. RNN can identify patterns of the network in time series and by default works on short-term memory cells. Memory term can be improved by LSTM, ensuring correlation between multiple time steps in a series. Gore, Padilla & Diallo (2017) applied Markov Chain techniques to the details of observed cyber threats to determine common vulnerabilities and develop proactive models. Musumeci et al. (2022) proposed an online DDoS attack detection method, by using KNN, ANN, support vector machine, and RF algorithms. Their proposed method achieved 98% accuracy in detecting SYN attacks. Ahuja et al. (2021) integrated random forest and support vector machine (SVM) methods for the classification of attacked and non-attacked traffic. Evaluation has been done on a dataset from a software-defined network (SDN) and achieved 98.8% accuracy with minimal false values. Tonkal et al. (2021) performed a comparative analysis by implementing multiple machine-learning methods for SDN data. Phan, Bao & Park (2016) implemented a hybrid method by integrating self organizing maps (SOM) and SVM, which improved the classification accuracy for DDoS flood attack detection against OpenFlow and SDN controllers. Swami, Dave & Ranga (2021) explored the random forest, MLP, logistic regression, LR, and DT for DDoS attack detection in an SDN using a network dataset. Nadeem et al. (2022) implemented naïve Bayes, support vector machine, KNN, and RF to detect the DDoS attack, and tested the implemented techniques using the NSL-KDD dataset and achieved 99.97% accuracy. Yuan, Li & Li (2017) sampled 20 network data features of the dataset ISCX 12 to design a bidirectional RNN performance analysis. Alkasassbeh et al. (2016) simulated DDoS attacks *via* HTTP flooding and SQL injection (SIDDOS) along with traditional UDP flooding and Smurf attacks. Application layer attacks, SIDDOS, and HTTP flooding have drawn more attention from researchers recently. NS2 network-based simulated dataset of multi-class with 1,048,575 instances is utilized to detect DDoS attacks namely SIDDOS, Smurf, UDP flooding, and HTTP flooding.

Literature analysis of DDoS detection in smart grid networks shows that the neural network-based DDoS detection strategies performed better than the others. However, deep autoencoders with reconstructive-based detection algorithms eliminating full model retraining requirements, have not yet been explored for DDoS attack detection. Therefore, we present an efficient reconstructive model-based scheme to bridge this research gap. In comparison to other machine learning-based techniques used for DDoS detection, the proposed performed better in terms of efficiency.

## Proposed Method

**Figure 1:**
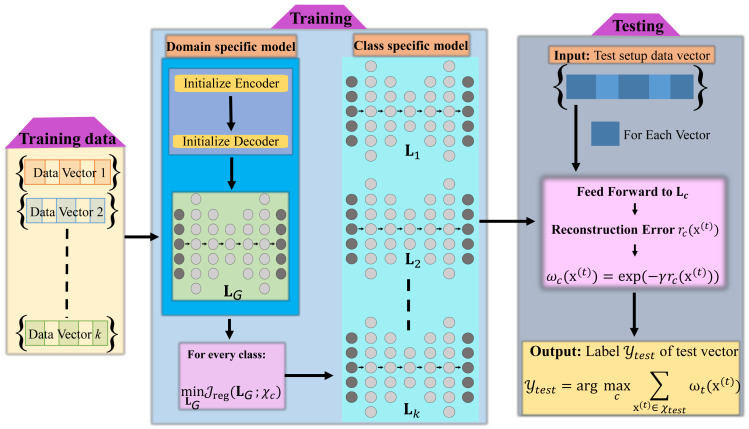
Block diagram of proposed reconstructive model-based attack classification algorithm.

This section contains complete details of the technique proposed for detecting DDoS attacks within smart grid networks. A complete framework of our proposed method, which leverages the reconstruction model is illustrated in Fig. 1. First, we briefly introduce the reconstructive models employed in our method. Next, we provide the details of our reconstruction error-based detection strategy. Specifically, we consider three types of models in our study. These include the deep autoencoder, the marginalized stacked denoising autoencoder, and the deep extreme learning machine-based autoencoder.

### Proposed class specific reconstruction based method for classification

The presented approach for DDoS attack detection based on reconstructive models consists of two primary stages. Firstly, we train a global domain-specific reconstructive model using the entire dataset in an unsupervised manner. In the second stage, we train class-specific reconstructive models by utilizing the global representation as an initial starting point. This methodology enables the encoding of both domain-specific and class-level data characteristics. Let $\mathbf{T}=\{ {\mathbf{X}}_{n}\} _{n=1}^{c}\in {\mathbb{R}}^{d\times N}$ be the training data for *c* attack classes having *N* samples: $N={\mathop{\sum }\nolimits }_{n=1}^{c}{s}_{n}$ in total, where *s*_*n*_ represents the count of training samples within the attack class denoted by index *n* which is defined as ${\mathbf{X}}_{n}=\{ {\mathbf{x}}_{n}^{i}\} _{i=1}^{{s}_{n}}\in {\mathbb{R}}^{d\times {s}_{n}}$ (where ${\mathbf{x}}_{n}^{i}\in {\mathbb{R}}^{d}$ is a *d*-dimensional feature vector. While the value of *s*_*n*_ may vary among different training classes, the dimension of ${\mathbf{x}}_{n}^{i}$ will remain consistent.Consider **Y** as the set of class labels, denoted as ${{y}_{n}}_{n=1}^{c}$, corresponding to the training classes within **T**. Our task of DDoS attack detection aims to predict the label *y*_*t*_ for the test sample **x**_*t*_ using the training dataset **T**.

***Training stage:*** During the training stage, we initiate the weights of a multi-layer neural network, forming a global domain-specific reconstructive model, using the autoencoder training. This model comprises *h* hidden layers and its parameters are acquired through an unsupervised manner utilizing all the available samples in **T**. We represent the global reconstructive model as **L**_*G*_, composed of its constituent parts ${\mathbf{W}}_{G}^{1},\ldots ,{\mathbf{W}}_{G}^{h+1}$. Each ${\mathbf{W}}_{G}^{i}$ corresponds to the parameter matrix of layer *i*, obtained using auto-encoding training techniques. This global reconstructive model serves as an initial framework, upon which we build class-specific reconstructive models in our subsequent stages.

As **L**_*G*_ encapsulates domain-specific representation, acquired through its training to reconstruct samples from that domain, we can employ it to build distinct reconstructive models for all the *c* training classes separately. That is, rather than starting with random initialization for the hidden layer weights, we preferred to employ the weights contained within **L**_*G*_ as the initial settings for our class-specific models. As a result, we establish a total of *c* reconstructive models, each tailored to one of the *c* classes, denoted as **L***jj* = 1^*c*^. Each class-specific model is explicitly defined as ${\mathbf{L}}_{j}={\mathbf{W}}_{j}^{1},\ldots ,{\mathbf{W}}_{j}^{h+1}$.

***Testing stage:*** During the testing stage, we reconstruct a given test sample ${\mathbf{x}}_{t}^{i}$ by applying each class-specific model from the set **L***jj* = 1^*c*^. The reconstructed sample obtained by the model **L***j* is represented as $\hat {\mathbf{x}}{tj}^{i}$ and can be defined as:

(1) \begin{eqnarray*}{\hat {\mathbf{x}}}_{tj}^{i}=f({\mathbf{x}}_{tj}^{i},{\mathbf{L}}_{j})={\mathbf{W}}_{j}^{h+1}g({\mathbf{W}}_{j}^{h},\ldots ,g({\mathbf{W}}_{j}^{1}{\mathbf{x}}_{t}^{i}))\end{eqnarray*}

where the reconstruction function is denoted as *f*, and *g* represents the activation function used by the layers. To quantify the reconstruction error for a given sample **x***t*^*i*^, we compute the distance (*e.g.*, Euclidean) between sample **x***t*^*i*^ and sample ${\hat {\mathbf{x}}}^{i}tj$, which is expressed as ${e}^{i}(j)={|}{\mathbf{x}}_{t}^{i}-{\hat {\mathbf{x}}}^{i}tj{{|}}_{2}$. Subsequently, the estimated label ${l}_{t}^{i}$ for ${\mathbf{x}}_{t}^{i}$ is determined by identifying the class associated with the minimum reconstruction error.

(2) \begin{eqnarray*}{l}_{t}^{i}=\arg ~\min _{j}~{\mathbf{e}}^{i}(j).\end{eqnarray*}

The entire process of the two stages discussed is outlined in Algorithm 1 .

 
_______________________ 
Algorithm 1 Proposed Reconstructive Model Based Attack Classification Al- 
gorithm_____________________________________________________________________________________________ 
Require: : 
  Training Dataset T containing c sets of attack samples Xs  = {xim}sm 
i=1  ∈ 
 Rd×sm grouped into c categories 
  Labels of classes denoted by Y = {ym}cm=1 
    Test sample xit ∈ Rd 
    Hidden layers h 
Ensure: : The predicted label yt for xt. 
Ensure: : Predicted Label yt for xt. 
  Training Phase: 
  Train Global Reconstructive Model LG = {W1G,...,Wh+1 
G   } using the entire 
   T and h hidden layers. 
  for j = 1 : c do 
      Train individual reconstructive models Lj  =  {W1j,...Wh+1 
j    } for each 
   class. 
  Testing Phase: 
  for j = 1 : c do 
      Reconstruct Test Sample: ˆ xitj = f(xit;Lj) {Reconstruction (??)} 
    Compute Reconstruction Error: ei(j) = ∥xit − ˆ xitj∥2 
    Predicted Label: lit ≜ arg min 
     j       ei(j)______________________________________________________    

### Different classes of autoencoders used for learning representations

An autoencoder is a neural network architecture trained *via* a back-propagation algorithm, wherein the input values are set equivalent to the target values, which blend into the input samples themselves. An autoencoder comprises two essential components: an encoder denoted as *h*(⋅), responsible for mapping an input data point **x**_*i*_ ∈ ℝ^*d*^ as a latent expression *h*(**x***i*) ∈ ℝ^*dh*^, further a decoder, referred to as *g*(⋅), tasked with reconstructing the input data **x**_*i*_ from this latent representation, ensuring that *g*(*h*(**x**_*i*_)) ≈ **x**_*i*_. The measure of this reconstruction is estimated using a predefined loss function *l*(**x**_*i*_, *g*(*h*(**x**_*i*_))), which quantifies the dissimilarity between the original data and its reconstructed part. The primary purpose is to minimize this loss function to adapt and optimize the parameters of the autoencoder. Various forms of loss functions are available, including but not confined to squared error loss or the Kullback–Leibler ($\mathcal{KL}$) divergence. In the field of machine learning literature, various types of autoencoders have previously been introduced (Rifai et al., 2011; Baldi & Hornik, 1989; Vincent et al., 2008; Williams & Hinton, 1986; Lee et al., 2009; Kavukcuoglu et al., 2009). In this article, we explore three types of autoencoders for our algorithm. These include the deep autoencoder, extreme learning machine autoencoder, and the marginalized denoising autoencoder. The encoders are explained briefly in the next sections.

#### Deep autoencoder

A deep autoencoder uses multiple layers of hidden units and is an efficient model for learning the underlying structure of the data. A deep autoencoder consists of a decoder and an encoder part with each having multiple hidden layers. The role of the encoder is to compute valuable representations from the input data. An encoder comprises a layers series interconnected by an activation function (non-linear) that converts the input data **x** into a representation **h**, as illustrated below:

(3) \begin{eqnarray*}\mathbf{h}=s({\mathbf{W}}_{e}^{(3)}{\mathbf{h}}_{2}+{\mathbf{b}}_{e}^{(3)}),{\mathbf{h}}_{2}=s({\mathbf{W}}_{e}^{(2)}{\mathbf{h}}_{1}+{\mathbf{b}}_{e}^{(2)}),{\mathbf{h}}_{1}=s({\mathbf{W}}_{e}^{(1)}\mathbf{x}+{\mathbf{b}}_{e}^{(1)}),\end{eqnarray*}

where ${\mathbf{W}}_{e}^{(i)}\in {\mathbb{R}}^{{d}_{i-1}\times {d}_{i}}$ is the encoder weight matrix for layer *i* with *d*_*i*_ nodes, ${\mathbf{b}}_{e}^{(i)}\in {\mathbb{R}}^{{d}_{i}}$ is the bias vector and *s*(⋅) is the element-wise non-linear activation function. In our case we use a sigmoid defined as $s(z)= \frac{1}{1+{e}^{-z}} $. The encoder parameters are obtained through a blended training method that involves the decoder and encoder. This combined training approach aims to reconstruct the input data while minimizing the specified cost function. The decoder consists of interconnected layers employing a non-linear activation function, tasked with input reconstructing **x** on behalf of the output **h** of the encoder. The resulting reconstruction, denoted as $\tilde {x}$, is described as:

(4) \begin{eqnarray*}\tilde {x}=s({\mathbf{W}}_{d}^{(3)}{\mathbf{x}}_{2}+{\mathbf{b}}_{d}^{(3)}),{\mathbf{x}}_{2}=s({\mathbf{W}}_{d}^{(2)}{\mathbf{x}}_{1}+{\mathbf{b}}_{d}^{(2)}),{\mathbf{x}}_{1}=s({\mathbf{W}}_{d}^{(1)}\mathbf{h}+{\mathbf{b}}_{d}^{(1)}).\end{eqnarray*}

Subsequently we will denote the model by $\mathbf{L}=\{ {\mathbf{W}}_{e}^{(i)},{\mathbf{W}}_{d}^{(i)},{\mathbf{b}}_{e}^{(i)},{\mathbf{b}}_{d}^{(i)}\} _{i=1}^{3}$. To ensure the best performance of autoencoder, it is necessary to initialize the weights appropriately. For this purpose, we initialize the weights of the encoder by performing layer-by-layer training through Gaussian restricted Boltzmann machines (RBM).

#### Marginalized denoising autoencoder

Denoising autoencoders function on the basic principle of purposefully introducing some corruption to the input samples into the hidden layer representation before mapping. This technique is used to enhance the learning capability of the model for learning robust and useful features among noisy or imperfect input data.

Their training process revolves around reconstructing the original input **x**_*i*_ from its corrupted counterpart $\tilde {{\mathbf{x}}_{i}}$ through the minimization of $l({\mathbf{x}}_{i},g(h(\tilde {{\mathbf{x}}_{i}})))$. Different types of data corruption are used in denoising autoencoders, including additive isotropic binary masking noise and Gaussian noise. Binary masking noise is a favored approach, by setting a part of features to zero within each input sample.

Vincent et al. (2010) designed stacked denoising autoencoders (SDA) by stacking multiple denoising autoencoders (MDAs), thereby boosting the learning of deep feature representations among multiple layers. However, SDAs (Vincent et al., 2010) come with particular limitations. Like, their training approach can be slow, mainly due to the utilization of a descent-based stochastic gradient and back-propagation algorithm. Further, SDAs implicate numerous hyperparameters like the mini-batch size, learning rate, noise ratio, number of epochs, and network structure, all of which necessitate tuning *via* verification data sets. This tuning process can further slow the training process. Additionally, non-convex optimization methodology and the selection of a suitable initialization method mainly affect the final outcomes.

Chen et al. (2012) presented an advanced variant of SDA termed marginalized SDA (MDA). MDA was developed to enhance training efficiency. One significant edge of MDA is that it facilitates a closed-form solution, eradicating the need for a back-propagation algorithm to optimize network parameters. This distinct induces MDA computations notably efficient in comparison to the traditional SDA. Similarly, the MDA technique delivers the flexibility of stacking multiple MDAs to construct deep feature representations than SDA. Notably, Chen et al. (2012) followed that MDA performance in terms of classification accuracy is better than SDA.

Inspired by the above benefits, this study leverages the MDA algorithm to achieve powerful feature representations for the aim of DDoS detection. The fundamental component of Marginalized Denoising Autoencoders (MDA) is a single-layer denoising autoencoder. From the training, data set $\mathbf{X}=\{ {\mathbf{x}}_{i}\} _{i=1}^{N}\in {\mathbb{R}}^{d\times N}$, Every input sample **x**_*i*_ is corrupted in random by extracting features (changing to 0). Particularly, a feature is assigned a weight of 0 with probability *p* ≥ 0. The MDA algorithm holds several advantages over traditional denoising autoencoders. Single iteration over data is One of the noticeable advantages which provides efficiency.

#### Extreme learning machine autoencoder

Efficiently obtaining rich data representations is of vital importance for acquiring strong generalization, specifically on a large scale. This task can commonly be achieved using autoencoders, where a parametric regressor function is learned to map input data to itself. While deep neural networks have shown great power across diverse learning tasks, they are often affected by slow training times. To tackle this problem, we utilize extreme learning machine-based autoencoders (ELM-AE) (Zheng et al., 2021) for unsupervised learning of dataset representations. ELMs are recognized for their computational efficiency during training. A deep ELM, basically a multi-layer neural network, learns its parameters *via* the training of multiple ELM layers. This learning technique excels in training speed while maintaining robust generalization abilities. In ELM-AE, the hidden nodes are provided with orthogonal random biases and weights, allowing them to project the input data into different or equal dimensions. DELM (Jiang, Zhang & Zhong, 2022) has demonstrated a high capability to effectively reveal the non-linear structure present in the data. Compared to deep neural networks, DELM reduces the need for resource-intensive iterative fine-tuning of weights.

## Experimental Results

We have conducted a comprehensive performance analysis of the proposed algorithm, with a primary emphasis on accuracy metrics. To make sure its significance, we used two benchmark datasets specifically for intrusion detection and conducted the comparative analysis of the obtained outcomes with other machine learning-based methods, decision-tree (Zekri et al., 2017), LSTM (Yuan, Li & Li, 2017), KN (Doshi, Apthorpe & Feamster, 2018), LSVM (Doshi, Apthorpe & Feamster, 2018), random forest (Yuan, Li & Li, 2017), and naive Bayes (Zekri et al., 2017). For a baseline comparison, we proposed a DDoS detection model using the ELM autoencoder. We then trained our proposed model on the unprocessed features, extracted from the labeled training data. Detailed insight of the datasets used for experimentations are:

### UNB ISCX Intrusion Detection Evaluation 2012 dataset

One of the datasets available publicly we incorporated for experimental analysis is the UNB ISCX Intrusion Detection Evaluation 2012 dataset (Ali & Li, 2019) referred to as IDE2012. We utilized two testbeds within this dataset. The first one is the testbed of 11th June, named IDE2012/11, and the second is the 16th June testbed referred to as IDE2012/16 as documented in UNB (2012). IDE2012/11 testbed contains 204 features each for 325,757 samples. IDE2012/16 testbed contains a total sample of nearly 464,989 and contains 204 features each. Labels provided for each sample are unrated, acceptable, safe, and unsafe. We further discretized these labels as unsafe (1), and safe (0) for simplification. Some of the significant attributes of each data packet are event generator, ndpi risk, Payload byte first, source and destination port, event priority and ndpi detected protocol, *etc*. 15% of this dataset (10,000 packets) demonstrated signs of DDoS attack infection. This observed infection rate serves to emphasize the prevalence and impact of DDoS attacks within the dataset. Part of this data set is shown in Table 2.

**Table 2:** Labels and features of four packets in IDE2012/11 dataset.

**src**	**payload**	**packet**	**dst**	**dst2src**	**src2dst**	**ndpi**	**ndpi**
**port**	**bytes first**	**header size**	**port**	**packet rate**	**packet rate**	**detection**	**risk**
1425	0	40	82	0	0	3	Unrated
4948	0	40	22	12	4	2	Acceptable
5585	0	46	109	913	406	1	Unsafe
64192	0	63	76	0	0	0	Safe

To assess the adaptability of our model among varying portions of data, we selected 10,000 samples randomly of every dataset. Additionally, extending beyond the traditional two classes 0 for safe and 1 for unsafe, our evaluation encompasses four-class classification settings. Designated labels are 0 for safe, 1 for unsafe, 2 for acceptable, and 3 for unrated. A breakdown of these dataset divisions along with names are shown in Table 3.

**Table 3:** Details of distinct subsets, packets, and features of IDE2012 dataset.

**Data**	**Subset**	**Total**	**Total**	**Labels**
**Subset**	**Named**	**Packets**	**Features**	
IDE2012/11	D1	325,757	204	0,1
IDE2012/11	D2	325,757	204	0,1,2,3
IDE2012/16	D3	464,989	204	0,1
IDE2012/16	D4	10,000	204	0,1
IDE2012/16	D5	464,989	204	0,1,2,3

### UNSW-NB15 dataset

One of the publicly available intrusion detection data set used in our experiments is UNSW-NB15 (UNSW, 2015) dataset. Australian university named The University of New South Wales (UNSW), created the data set utilizing the IXIA perfect-storm tool and offers extensive network data of 700,001 samples with 49 features each, captured under a controlled environment. To push the limits of the proposed method, we further partitioned the UNSW-NB15 dataset into four distinct subsets are shown in Table 4.

**Table 4:** Details of distinct subsets, features and packets of UNSW-NB15 dataset.

**Data**	**Subset**	**Total**	**Total**	**Labels**
**Subset**	**Named**	**Packets**	**Features**	
UNSW-NB15-1	D6	700,001	49	0, 1
UNSW-NB15-2	D7	700,001	49	0, 1
UNSW-NB15-3	D8	700,001	46	0, 1
UNSW-NB15-4	D9	440,044	46	0, 1

A few features of the UNSW-NB15 dataset are attack category, source IP, transaction protocol, record start time, destination IP, source jitters (mSec), duration, *etc*. For experimentation, we transformed non-numerical features into discrete features. We further discretized the provided label and assigned “attack” a value of 0 and “non-attack” a value of 1.

## Experimental Setup

We have a total of nine datasets renamed D1 to D9. For evaluation of the proposed method, we divided each dataset randomly to 20% for testing purposes and 80% for training purposes. The experiments were repeated nine to ten times, and the average accuracy was calculated on the basis of the outcomes of these experiments. Performance indicators employed for the evaluation of the proposed method encompass *FP* (false positive), *TN* (true negative), *FN* (false negative), and *TP* (true positive). Where, *FN* indicates legitimate data detected as abnormal, *TN* indicates legitimate data seen as legitimate, *FP* indicates the abnormal data detected as legitimate, and *TP* indicates the abnormal data correctly identified as abnormal. These metrics are utilized in the calculation of *Accuracy* $= \frac{TP+TN}{FN+FP+TP+TN} $ of the proposed method. The proposed method contains parameters from MSDA. The fundamental parameters operated for feature learning are the total layers in every MSDA (*L*), total deep MSDAs (*M*), and corruption probability (*p*). For experiments, we denoted the total layers in every MSDA as *L*_*m*_ and assigned between [1, 3, 5, 7, 9, 11], (*M*) to nine MSDAs, and the corruption probability denoted as *p*_*m*_ and chosen between 0.1, 0.2, …, 0.5. These parameters can undergo additional fine-tuning within a cross-validation framework to potentially improve results. To ensure optimal performance, we compared the algorithms using the recommended parameters from the original authors. For evaluation, we employed a 10-fold cross-validation technique for available datasets and their sub-datasets. The conclusive detection accuracy is calculated as the standard of these 10-fold. These experiments were conducted in MATLAB on a computer with NVIDIA Tesla V100 GPUs and 32.5 GB of memory.

## Conclusions and Evaluation

**Figure 2:**
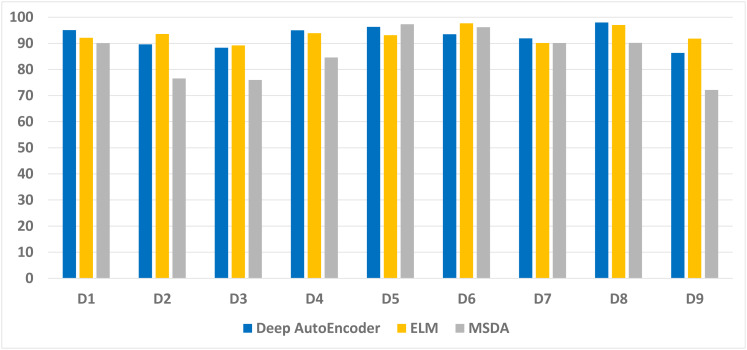
Average accuracy achieved by our technique on the nine data sets.

Figure 2 illustrates the average accuracy attained by the Deep autoencoder, ELM, and MSDA on the nine data sets. The accuracy on the large data sets is slightly lower than the accuracy achieved on the smaller data sets. It Happened because the total test set of these data sets is larger. Hence, the proposed method learned good features from larger and smaller data sets equally.

Table 5 presents a comprehensive evaluation of the performance of an extreme learning machine (ELM)-based classification model across nine datasets (D1-D9). Precision, recall, specificity, F1 score, and area under the receiver operating characteristic curve (AUC) are reported for each dataset, providing insights into the model’s discriminative capabilities, sensitivity to positive instances, ability to correctly identify negative instances, overall precision–recall balance, and the area under the ROC curve. The average values, accompanied by standard deviations, show a detailed view of the model’s consistency in performance.

**Table 5:** Average and standard deviations of the different performance metrics of the proposed method on D1 to D9 Datasets for 10-fold experiments.

**Datasets**	**Precision**	**Recall**	**Specificity**	**F1 score**	**AUC**
D1	0.92 ± 0.01	0.93 ± 0.02	0.90 ± 0.01	0.92 ± 0.01	0.95 ± 0.005
D2	0.96 ± 0.02	0.98 ± 0.01	0.95 ± 0.02	0.97 ± 0.01	0.98 ± 0.008
D3	0.89 ± 0.01	0.91 ± 0.02	0.88 ± 0.01	0.90 ± 0.02	0.94 ± 0.006
D4	0.94 ± 0.01	0.96 ± 0.01	0.93 ± 0.01	0.95 ± 0.01	0.97 ± 0.007
D5	0.89 ± 0.02	0.91 ± 0.02	0.88 ± 0.02	0.90 ± 0.02	0.94 ± 0.006
D6	0.95 ± 0.01	0.96 ± 0.01	0.93 ± 0.01	0.95 ± 0.01	0.97 ± 0.007
D7	0.92 ± 0.01	0.92 ± 0.02	0.90 ± 0.01	0.92 ± 0.01	0.95 ± 0.005
D8	0.93 ± 0.02	0.95 ± 0.01	0.92 ± 0.02	0.94 ± 0.01	0.96 ± 0.006
D9	0.88 ± 0.02	0.90 ± 0.02	0.97 ± 0.02	0.89 ± 0.02	0.93 ± 0.007
Average	0.92 ± 0.01	0.93 ± 0.01	0.91 ± 0.01	0.92 ± 0.01	0.95 ± 0.006

**Figure 3:**
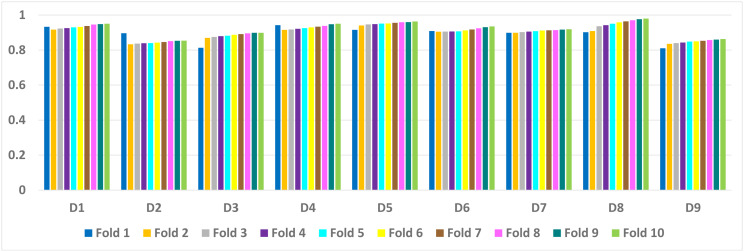
AutoEncoder: 10 fold experimental results of D1 to D9 data sets.

**Figure 4:**
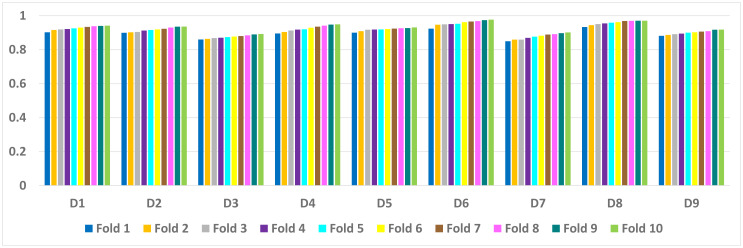
ELM: 10 fold experimental results of D1 to D9 data sets.

**Figure 5:**
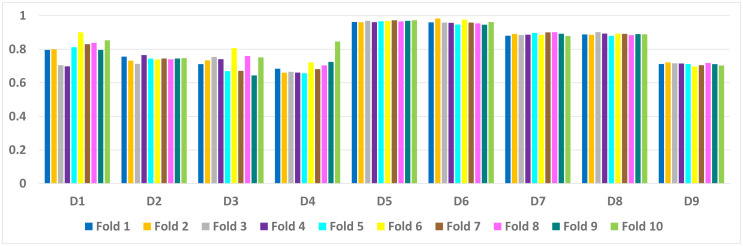
MSDA: 10 fold experimental results of D1 to D9 data sets.

Table 6 presents a detailed comparison of the accuracy of the proposed algorithm with machine learning-based other six DDoS attack detection techniques. The proposed algorithm has performed much better than the other methods. Because our method learned multiple autoencoders and the proposed reconstructive-based scheme for classification. Also, the use of deep extreme machine learning-based autoencoder enabled us to learn useful features from training data. LSTM (Yuan, Li & Li, 2017) performed better than the rest of the previously used machine learning-based methods. However, the proposed method achieved more good outcomes than LSTM based method because of the fusion of the proposed deep reconstructive machine learning-based classification. 10 fold experimental results of Deep AutoEncoder, ELM, and MSDA on dataset D1 to D9 are shown in (Figs. 3, 4 and 5).

**Table 6:** Performance metrics and variability from 10-fold cross-validation experiments.

Data	LSTM	Random forest	Naive-Bayes	Decision-tree	KNN	LSVM	**Proposed**
sets	Yuan, Li & Li (2017)	Yuan, Li & Li (2017)	Zekri et al. (2017)	Zekri et al. (2017)	Doshi, Apthorpe & Feamster (2018)	Doshi, Apthorpe & Feamster (2018)	
D1	90.5 ± 1.3	89.7 ± 1.7	88.1 ± 2.3	89.9 ± 1.6	90.0 ± 1.9	90.1 ± 2.5	91.0 ± 2.0
D2	96.3 ± 2.1	95.2 ± 2.1	93.6 ± 1.6	95.8 ± 1.3	95.9 ± 2.3	92.8 ± 1.9	97.0 ± 2.8
D3	88.7 ± 1.5	86.1 ± 1.8	88.5 ± 1.2	87.3 ± 1.1	88.1 ± 1.5	86.4 ± 2.0	90.0 ± 1.9
D4	94.7 ± 1.9	89.8 ± 1.3	93.3 ± 1.0	94.1 ± 1.4	92.3 ± 2.8	90.5 ± 1.9	95.5 ± 2.7
D5	89.1 ± 2.0	88.2 ± 1.8	87.5 ± 1.1	86.8 ± 2.2	88.3 ± 1.9	85.5 ± 1.5	90.0 ± 1.1
D6	94.2 ± 1.1	93.3 ± 2.1	92.6 ± 0.7	91.8 ± 1.7	93.4 ± 2.5	89.7 ± 1.9	95.5 ± 2.7
D7	90.2 ± 2.0	88.2 ± 1.7	85.5 ± 1.2	87.8 ± 1.3	84.7 ± 1.9	88.3 ± 2.3	91.0 ± 2.0
D8	93.6 ± 1.3	87.7 ± 1.9	90.2 ± 1.7	89.5 ± 2.1	93.3 ± 1.5	90.2 ± 2.8	94.0 ± 2.3
D9	88.7 ± 1.6	86.7 ± 1.1	88.1 ± 1.2	84.5 ± 1.6	79.3 ± 2.3	82.3 ± 2.5	90.0 ± 1.9
Average	92.8 ± 1.6	88.5 ± 1.6	89.4 ± 1.5	89.2 ± 1.6	88.7 ± 2.2	88.0 ± 2.1	93.0 ± 2.2

### Impact of number of neurons over accuracy

We have evaluated the accuracy of the proposed technique by integrating varying numbers of Neurons to construct the ultimate classifier. The datasets used for experimentations are D1 to D9. Precisely, we changed the amount of Neurons to be combined, between 2 to 10. Furthermore, we randomly selected the number of layers for each Neuron from the set *L*_*m*_ = [1, 3, 5, 7, 9, 11]. Subsequently, these Multi-layer Stacked Denoising Autoencoders (MSDAs) are fused using the Reconstructive-based scheme for classification, resulting in the creation of our final classifier.

**Figure 6:**
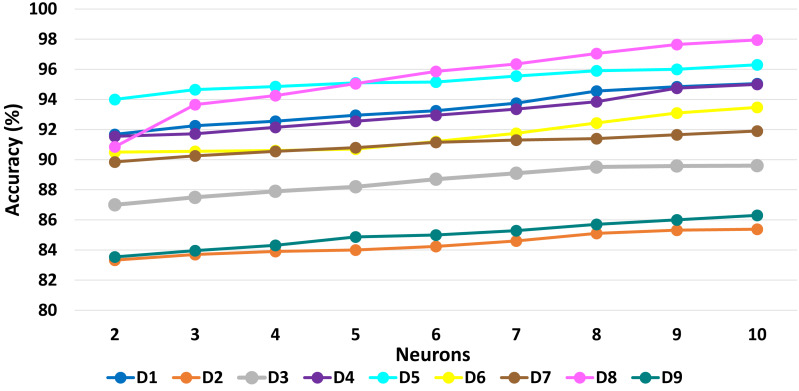
AutoEncoder: Impact of varying neuron numbers on accuracy across datasets D1 to D9.

**Figure 7:**
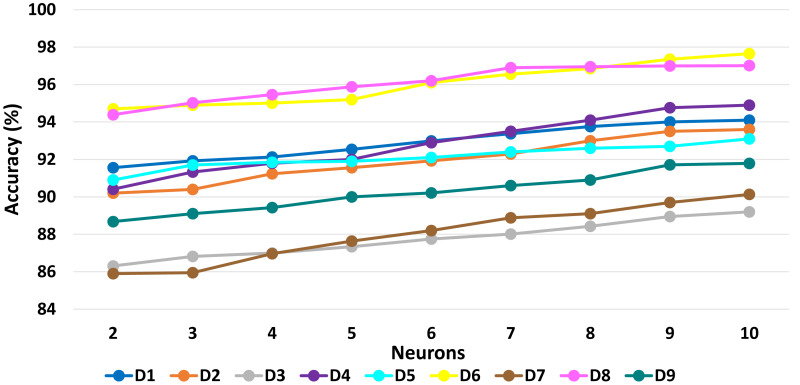
ELM: Impact of varying neuron numbers on accuracy across datasets D1 to D9.

**Figure 8:**
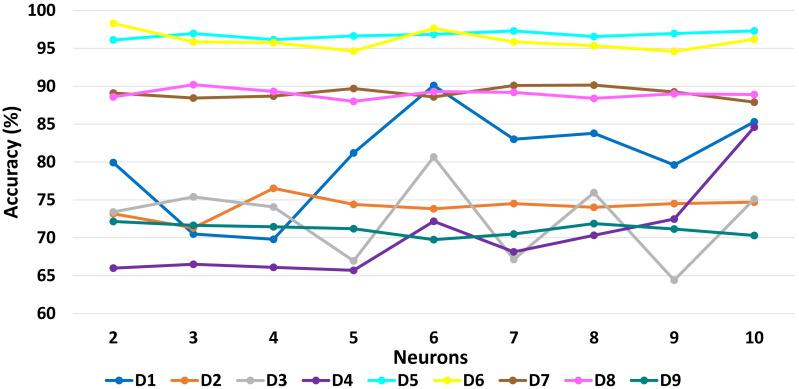
MSDA: Impact of varying neuron numbers on accuracy across datasets D1 to D9.

The accuracy results presented in Figs. 6, 7, 8 show the variation in performance by varying numbers of MSDAs for each dataset. Eventually, the accuracy improved with the increment of MSDAs.Evidently, as the number of MSDAs increases, the accuracy improves across all datasets and starts saturating at approximately six MSDAs. This trend underscores the effectiveness of the proposed fusion approach.

## Conclusion

Interconnection of communication devices from customers to a grid can make it more vulnerable to network attacks. With the rapid development of smart grids, it is crucial to have cutting-edge and adaptable solutions to guarantee their secure and efficient functioning in an increasingly interconnected world. We proposed a reconstructive deep-learning approach that can easily integrate any new class of attack without the need to retrain the whole system. The proposed method includes training or shallow and deep reconstructive models, each geared towards understanding and classifying different attack types. A novel class-specific reconstructive error-based classification underpinned the accurate attack identification. Experiments are conducted using two intrusion detection datasets and their subsets. The outcomes revealed that the proposed approach performed better than the other approaches under consideration in the aspect of accuracy. The uniqueness of the proposed method is its capability to learn new attack classes without any retraining, making it different from conventional methods and minimizing the possibility of disruption in the regular operation of the smart grid.

## Supplemental Information

10.7717/peerj-cs.1784/supp-1 Supplemental Information 1 Matlab code for ELM_AE_Reconstruct

10.7717/peerj-cs.1784/supp-2 Supplemental Information 2 Matlab code for ELM_AE

10.7717/peerj-cs.1784/supp-3 Supplemental Information 3 Matlab code for procrustNew

10.7717/peerj-cs.1784/supp-4 Supplemental Information 4 Matlab code and other required codes

## Additional Information and Declarations

### Data Availability

The following information was supplied regarding data availability: The code is available in the Supplemental Files.
